# Transient global amnesia after the right temporal epilepsy surgery: A case report

**DOI:** 10.1002/epi4.13009

**Published:** 2024-07-06

**Authors:** Elisa Schütz, Dirk‐Matthias Altenmüller, Kathrin Wagner, Marcel Heers, Andreas Schulze‐Bonhage, Birgitta Metternich

**Affiliations:** ^1^ Epilepsy Center, Department for Neurosurgery, Medical Center University of Freiburg Freiburg Germany

**Keywords:** epilepsy, epilepsy surgery, neuropsychology, temporal lobe epilepsy, transient global amnesia

## Abstract

Transient global amnesia (TGA) is characterized by sudden and temporary memory impairment, while transient epileptic amnesia (TEA) represents amnestic attacks as the main manifestation of focal epilepsy with presumed temporal origin. We present a 48‐year‐old patient who experienced transient amnesia 10 weeks after right selective amygdalo‐hippocampectomy for right temporal lobe epilepsy. Despite TEA being a plausible explanation for amnesia in patients with temporal lobe epilepsy, no epileptiform discharges were found during the amnestic episode and key features indicative of TGA, including long duration, isolated occurrence, and dense anterograde amnesia of the episode, argued against a diagnosis of TEA in this case. Notably, the patient has remained seizure‐free (now 4,5 years) and stopped taking antiseizure medication 32 months after surgery. Although TGA clinical criteria formally exclude patients with recent active epilepsy, neurologists should be aware that TGA can occur after epilepsy surgery in the temporal lobe. Therefore, we consider it of high clinical relevance to establish a careful differential diagnosis between TGA and epileptic amnestic attacks after epilepsy surgery to avoid unnecessary reintroduction or continuation of antiseizure medication. Additionally, this case presents the first comparison of detailed neuropsychological test results before and after a presumed TGA episode, revealing a complete recovery of anterograde memory functions within 1 day.

**Plain Language Summary:**

A 48‐year‐old patient experienced an episode of transient amnesia 10 weeks after epilepsy surgery. Given the patient's history, an epileptic origin of the episode initially seemed likely. However, tests revealed no seizure activity during the episode and the characteristics matched a condition called transient global amnesia. This case highlights the importance of correctly diagnosing memory impairments after epilepsy surgery to prevent unnecessary treatment.

**Key Points**

- A male epilepsy patient experienced transient global amnesia ten weeks after right amygdalo‐hippocampectomy.
- This case highlights the need for a differential diagnosis between transient global amnesia and epileptic amnestic attacks after epilepsy surgery.
- Neuropsychological assessments before and after the episode revealed largely normal and stable cognitive functions.

## 1 INTRODUCTION

*Transient global amnesia* (TGA) is characterized by an abrupt and temporary (<24 h) disruption of anterograde memory, which results in patients typically repeating the same questions or statements.^1^, ^2^, ^3^, ^4^ Based on the clinical picture, a transient dysfunction of memory‐eloquent brain structures, mainly involving the medial temporal lobe, seems to be a common pathophysiological correlate. Indeed, PET and SPECT analyses indicate hypoperfusion in those areas in the acute stage. Studies using diffusion‐weighted MRI (DWI) further revealed that approximately 50% of the patients show reversible hyperintensities in the hippocampus during or shortly after acute events.^5^, ^6^, ^7^, ^8^ In some,^9^ but not all,^5^ MRI studies, hippocampal lesions were visible in T2 weighting even after a few months.

As a differential diagnosis (see Table 1 for comparison), *transient epileptic amnesia* (TEA) refers to amnestic attacks as the sole or main manifestation of seizures in temporal lobe epilepsy (TLE). The diagnostic criteria, as proposed by Zeman et al. (1998), include recurrent witnessed episodes of transient amnesia with otherwise intact cognitive functions and evidence supporting an epilepsy diagnosis, such as epileptiform abnormalities on EEG, the concurrent onset of other epilepsy features, or a clear‐cut response to anticonvulsant therapy. Compared to TGA, episodes of TEA are typically briefer, often occurring upon waking, and have a high recurrence rate. When available, ictal EEG recordings may show seizure activity involving both temporal lobes.^10^, ^11^, ^12^

**TABLE 1:** Comparison of characteristics of TGA and transient epileptic amnesia.

	TGA	TEA	Case
History of epilepsy	No	Yes	Yes
EEG abnormalities	No	Often bitemporal	Right temporal (non‐specific slowing, consistent with epilepsy surgery)
MRI	Hippocampal hyperintensity (DWI; in some cases)	Mild bilateral hippocampal atrophy	Resection defect after right sAHE
Age (years)	Typically 50+	Typically 50+	48
Duration of the episode	<24 h (typically 4–6 h)	Typically <1 h	6.5 h
Subsequent recollection of the episode	No	Usually	No
Recurrence rate	Low (6%–10% recurrence rate/year)	High (around 1/month)	No
Precipitants	Physical/psychological stress	Waking	Waking
Focal neurological or cognitive deficit (excluding amnesia)	No	No	No
Other features during the episode (sometimes present)	Headache, nausea	Semiological features of temporal lobe epilepsy (e.g., automatisms)	No
Ictal memory			
Anterograde amnesia	Yes	Variable	Yes
Retrograde amnesia	Variable	Often	Partial
Post‐ictal anterograde memory	Unimpaired	Unimpaired	Unimpaired^a^
Post‐ictal remote memory	Unimpaired	Frequent autobiographical and topographical memory deficits	Unimpaired

*Note*: References: Baker et al.,^10^ Butler & Zeman,^15^ Hodges and Warlow,^4^ Lanzone et al.,^16^ Sander et al.,^2^ Zeman et al.^12^

Abbreviations: DWI, diffusion weighted imaging; sAHE, selective amygdalo‐hippocampectomy; TEA, transient epileptic amnesia; TGA, transient global amnesia.

^a^ Diagnosticum für Cerebralschädigung (DCS) &Verbal Learning and Memory Test (VLMT, German version of the AVLT = Auditory Verbal Learning Test), both tests conducted 21 h after the amnesia onset.

When amnestic episodes occur in patients previously diagnosed with TLE, a causal relationship with temporal seizures may initially appear plausible. However, the situation becomes more complex when a patient has received surgical treatment for TLE. In this report, we present the case of a patient who experienced an episode of transient amnesia about 2.5 months after undergoing epilepsy surgery within the right temporal lobe.

## 2 CASE REPORT

A 48‐year‐old patient with late‐onset (44 years) medically refractory right TLE, arterial hypertension and congenital heart insufficiency underwent a right‐sided selective amygdalo‐hippocampectomy (sAHE) after invasive presurgical evaluation. The results of the bitemporal invasive stereo‐electroencephalography of this patient have been published separately.^13^ Before surgery, he experienced seizures starting with aura of epigastric sensation followed by déjà‐vu, impaired awareness, seizures with oral automatisms, and dizziness, with no signs of post‐ictal amnesia. He had recurrent depressive episodes, with no history of migraine or recent head injury.

**FIGURE 1:**
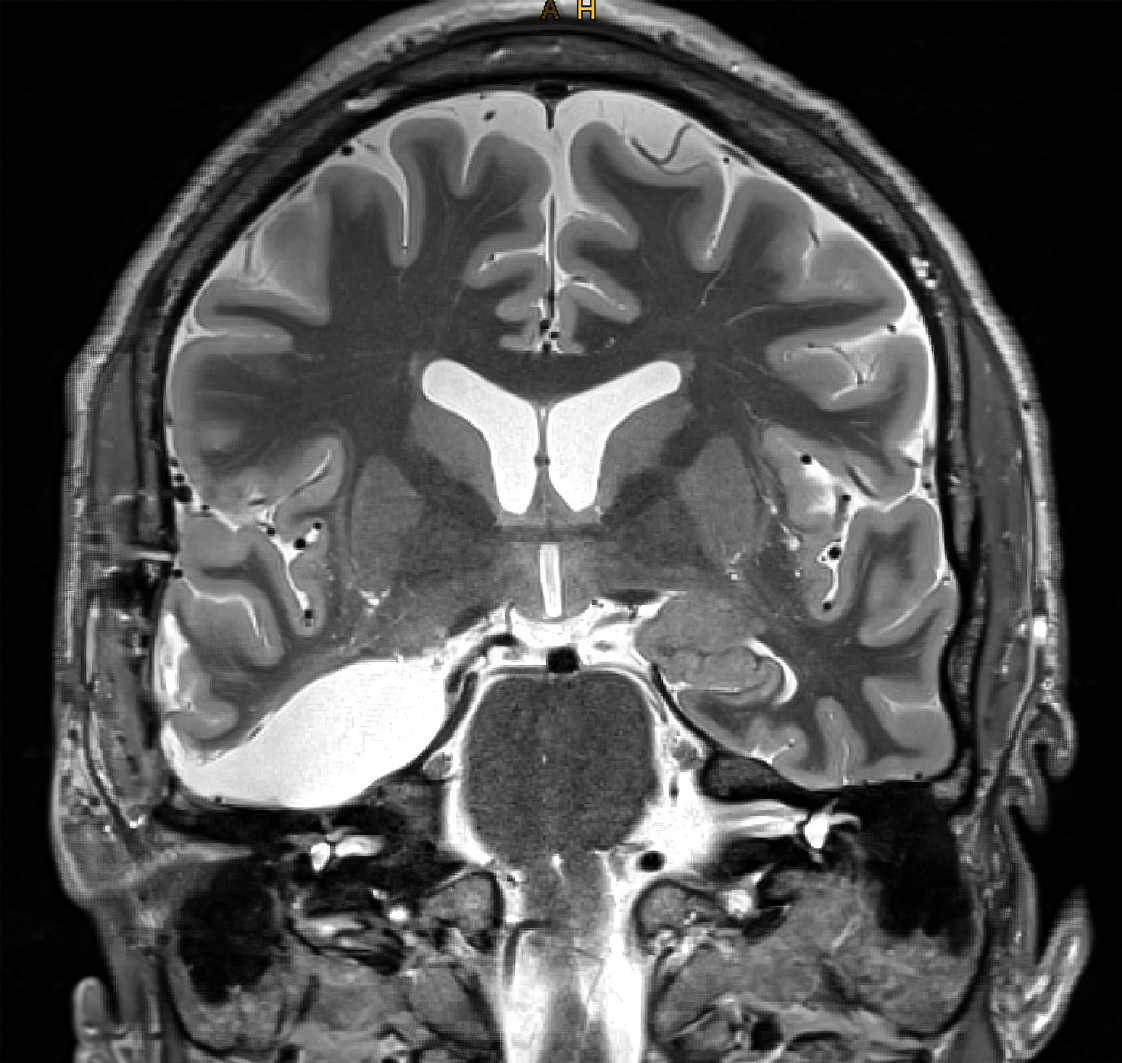
Coronal short‐time inversion recovery sequence of a 3‐Tesla MRI 4 months after surgery/6 weeks after an episode of TGA showing the status after right‐sided selective amygdala‐hippocampectomy.

An extensive neuropsychological assessment revealed results within the normal range, apart from slightly impaired verbal short‐term and working memory and non‐verbal working memory (see Appendix 1). Long‐term anterograde memory was intact. Functional neuroimaging showed a left hemispheric language lateralization. After surgery, he displayed no signs of surgical complications and reported no memory complaints. A postoperative MRI showed normal findings consistent with the performed surgery, without any evidence of edema or similar abnormalities (see Figure 1). Histopathological evaluation of the resected specimen revealed pronounced gliotic changes consistent with hippocampal gliosis, but no evidence of sclerosis or limbic encephalitis. The patient has remained seizure‐free (Engel Outcome 1a, follow‐up: now 4.5 years) and stopped taking antiseizure medication 32 months after surgery. Repeated EEG evaluations (the most recent one performed 45 months after surgery) revealed no epileptiform discharges.

Approximately 2.5 months after epilepsy surgery, the patient experienced an amnestic episode lasting 6.5 h. That day, at three o'clock in the afternoon, he slept and approximately an hour later, upon awakening, his partner noticed that he was asking strange and repetitive questions indicating memory loss. They visited the office of his outpatient neurologist, where an EEG was recorded (see Appendix 2), and went to the emergency room. Apart from the repeated questions (indicating an anterograde memory disorder for content >2 min), the patient reacted appropriately during the amnestic episode, e.g., being able to pack his suitcase. He exhibited disorientation to time and place, but not person, and remained conscious and able to communicate. There were also no apparent deficits in his autobiographical memory, extending beyond a few days before the episode (partial retrograde amnesia), nor any problems with semantic memory. After the episode, which lasted until about 10:30 p.m., the patient reported subjective well‐being. He had no memory of the event itself and his recollection of the hours leading up to the episode was severely fragmented.

Apart from the memory impairment, clinical examination revealed no focal neurological deficit. The patient was taking his antiseizure medication (lacosamide, perampanel) in the same dosage as before surgery. The EEG during the episode showed no epileptiform discharges (Appendix 2). An additional EEG conducted at the hospital, 12 h after amnesia onset, showed intermittent right temporal slowing reflecting the preceding sAHE on the right. No EEG pathology was identified in the left hemisphere. A cranial CT scan shortly after the episode (11 p.m.) ruled out hemorrhage or infarction and showed the expected resection cavity in the mesial temporal lobe. Additionally, high‐resolution 3 T MRI scans including DWI sequence taken one and 2 days after the episode, revealed no punctiform hippocampal lesions as reported after a TGA,^5^, ^6^, ^7^, ^8^ which fell within the timeframe to achieve the maximum diagnostic yield of DWI in TGA patients, which has been shown to be 24 to 96 h after symptom onset.^14^ From an epileptological view, a causal relationship between the amnestic episode and a recurrence or persistence of epilepsy or the surgical intervention could not be established.

**FIGURE 2:**
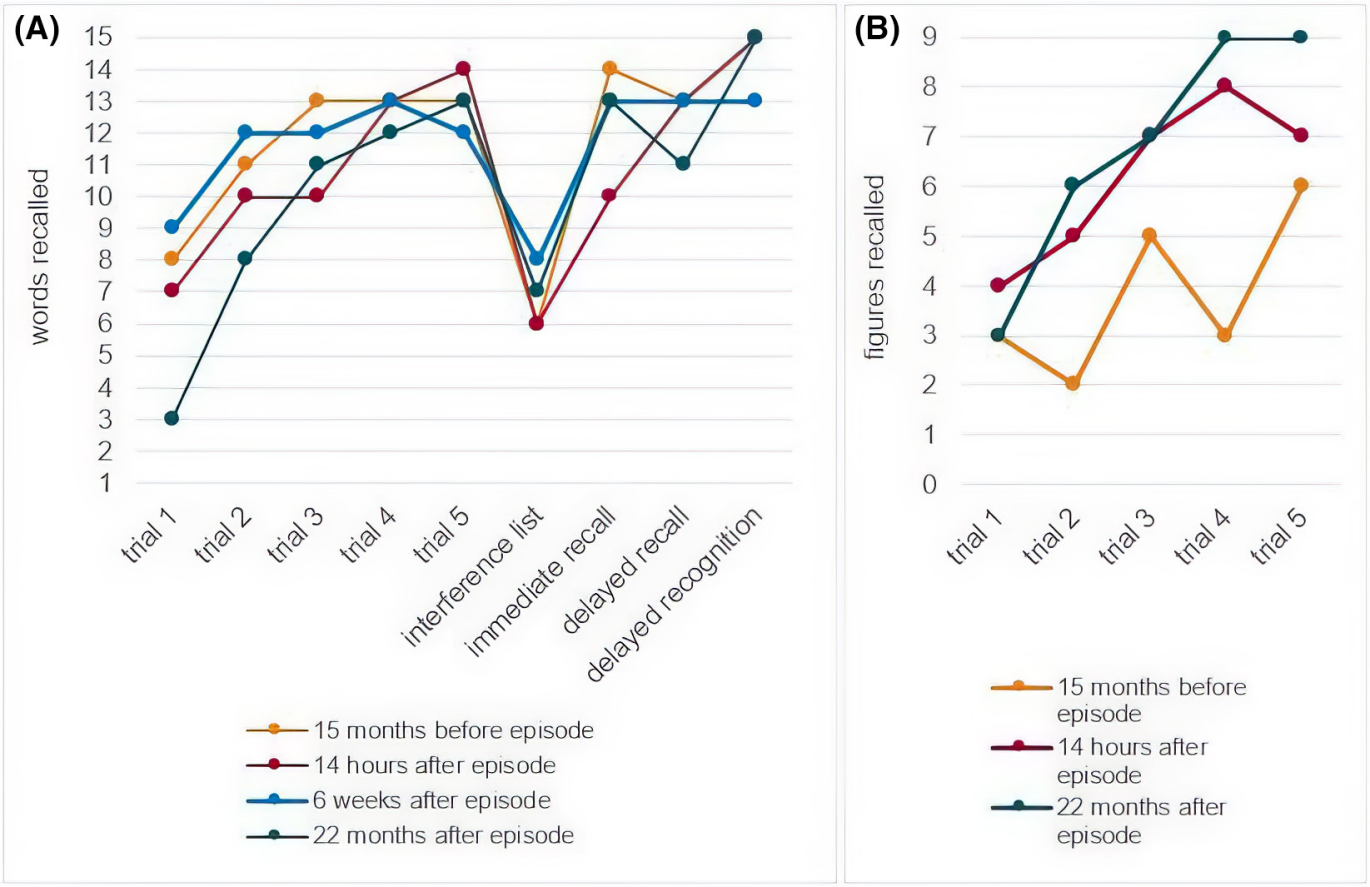
Verbal memory (A) and visual memory (B) before and after the episode. Note: Verbal Learning and Memory Test (German version of the AVLT = Auditory Verbal Learning Test). “Diagnosticum für Cerebralschaedigung.” Six weeks after the episode, only verbal memory was assessed.

The next day, about 14 h after the end of the episode, a brief neuropsychological assessment was performed (for details, see Appendix 1). Overall, there was only a slight increase in retroactive interference in word list learning compared to the presurgical evaluation, with otherwise unchanged and unimpaired verbal and non‐verbal memory performance. In the Mini‐Mental Status Examination (MMSE), the patient scored 29 out of 30 points. About 6 weeks later, a third and more comprehensive neuropsychological examination was conducted. Subjectively, the patient did not notice any cognitive decline, which was supported by the test results, showing mostly unchanged and unimpaired cognitive abilities. The previously observed tendency towards retroactive interference was no longer detectable. However, the intrusion rate was somewhat higher compared to the preoperative assessment. Two years after the surgery, there was a significant decline in verbal learning performance (see Figure 2A) and figural fluency, though performance in both tests still fell within the normal range. Other cognitive aspects, including non‐verbal memory (see Figure 2B), remained unchanged or improved (cognitive flexibility, TMT‐B) compared to the presurgical examination.

## 3 DISCUSSION

In 1990, Hodges and Warlow^4^ reported 114 cases with acute transient amnesia and established the clinical diagnostic criteria for TGA, which are still used today. One of these criteria is the exclusion of patients with recent active epilepsy (i.e., remaining on medication or having experienced a seizure in the past 2 years). Therefore, TEA seems to be a plausible explanation for transient amnesia in patients previously diagnosed with TLE and taking antiseizure medication, as seen in our case. Additionally, the episode occurring shortly after waking up aligns with what is commonly reported in TEA. While most TEA episodes documented in the literature were brief (<1 h), there have been instances of longer episodes, suggesting a prolonged post‐ictal state characterized by amnesia and confusion in these patients. Specifically, amnesia upon waking, as observed in our case, may indicate persistent post‐ictal dysfunction of medial temporal lobe structures following a seizure during sleep.^15^, ^16^ The use of a 24‐h EEG would have been beneficial to detect any interictal epileptic abnormalities during sleep.^17^, ^18^

However, some factors in our case argue against a diagnosis of TEA in this patient (see Table 1). Most importantly, the EEG conducted during the amnestic episode revealed no epileptiform discharges and the patient did not exhibit any other symptoms indicative of epileptic seizures. After undergoing successful right‐temporal epilepsy surgery, this amnestic episode was a singular occurrence and clearly differed from the seizure semiology he had shown before surgery. He remained seizure‐free with repeated normal EEG recordings. Another point is the type of memory impairments which aligned more with what has been reported for TGA. For example, we observed dense anterograde amnesia with no apparent deficits in autobiographical memory. In TEA, repetitive questioning (indicative of anterograde amnesia) is less observed compared to TGA but retrograde amnesia is much more prevalent, with severe deficits in autobiographical memory across the entire lifespan. Another crucial aspect of TGA is that it primarily affects episodic memory, while semantic memory remains largely intact, which was also observed in our case.^12^, ^15^, ^19^

Although the exact etiology of TGA remains unknown (for overview see^20^), there is consistent evidence pointing to a transient disturbance of hippocampal circuits involved in memory processing. Additionally, structural or diffusion‐weighted MRI and SPECT analyses have suggested a direct involvement of the hippocampus in TGA. In our case, the absence of one hippocampus after complete hippocampectomy may mean that any venous or other insult to the remaining hippocampus could more easily result in a severe and global amnestic state. Indeed, Dupont et al.^21^ reported typical episodes of TGA in five patients, each occurring several years after anterior temporal lobectomy including complete removal of the hippocampus (right: 4, left: 1) for refractory mesial TLE. These five patients bear similarities to our case: All patients experienced a single, typical TGA episode characterized by sudden onset, a temporary loss of anterograde and recent retrograde memory, and a duration of several hours. All patients were considered seizure free. Neither an epileptic nor vascular origin could be proven. All patients were younger than the classical TGA population, with the youngest being 36 years old. A key difference from our case is that the TGA episodes occurred between 3 and 12 years after surgery, whereas our patient underwent surgery only 2.5 months prior to the episode. While our single observation does not allow us to establish a definite causal relationship between the amnestic attack and the preceding surgery, the presence of similar cases suggests that hippocampal removal may potentially act as a facilitating factor for TGA.

There are some accounts of cognitive deficits after TGA episodes. However, in all studies to date, it was not possible to distinguish whether these deficits are a direct result of the episode itself or if they were preexistent. In our case, the routine neuropsychological testing conducted before epilepsy surgery provided a unique opportunity to compare cognitive performance before and after a suspected TGA episode. Fourteen hours after the episode, we observed completely normal and unchanged declarative memory in our patient, despite having undergone sAHE, which carries the risk for memory decline. Although some evidence suggests that neuropsychological deficits may persist for more than 24 h in a small subgroup of patients,^22^, ^23^ our findings are in accordance with the majority of cases described in the literature, where normal memory performance was observed once the TGA episode subsided.^4^ Moreover, both short‐term (6 weeks) and long‐term (2 years) cognitive functions remained largely unchanged, except for a significant decline in verbal learning and figural fluency after 2 years. This decline is unlikely to be related to the TGA episode or the surgery, as no significant changes were observed in the testing conducted a few hours after the episode or 3 months after surgery. Furthermore, performance in both tests was still within the normal range at the final assessment.

## 4 CONCLUSION

The clinical criteria of TGA formally exclude patients with recent active epilepsy.^4^ However, our case and similar cases from the literature suggest that TGA may occur, even if rarely, after surgery in the mesial temporal lobe. This warrants a clear differential diagnosis between TGA and epileptic amnestic attacks especially in patients who have undergone epilepsy surgery and became seizure‐free. Misdiagnosing TGA as an epilepsy related amnestic attack could result in the unnecessary reintroduction or continuation of anti‐seizure medications, potentially causing adverse psychological and socio‐economic consequences.

## CONFLICT OF INTEREST STATEMENT

None of the authors has any conflict of interest to disclose. We confirm that we have read the Journal's position on issues involved in ethical publication and affirm that this report is consistent with those guidelines.

## Data Availability

Data sharing not applicable to this article as no datasets were generated during the current study.

## APPENDIX 1 COGNITIVE PERFORMANCE

### 1.1

**Table:**

	15 months before episode/13 months prior to surgery	14 h after episode/2.5 months after the epilepsy surgery	6 weeks after episode/4 months after the epilepsy surgery	21.5 months after episode/24 months after the epilepsy surgery
Cognitive Screening & Executive Functions				
MMSE (raw score)	—	29	—	—
Trail making test‐B (time in seconds)	64	—	59	38
RWT Category fluency (words/2 min)	40	—	—	34
RWT Letter fluency (words/2 min)	9	—	—	8
Five‐point test (figures/3 min)	38	—	—	26
Speed of information processing				
Trail making test‐A (time in seconds)	27	—	23	22
Short‐term and working memory				
WMS‐R Digit span forward (raw score)	**6**	—	7	**6**
WMS‐R Digit span backward (raw score)	**5**	—	6	**5**
WMS‐R Visual span forward (raw score)	9	—	7	9
WMS‐R Visual span backward (raw score)	**6**	—	9	10
Long‐term memory				
VLMT verbal learning (sum of trials 1–5)	58	54	58	47
VLMT early recall (words after 20–30 min)	13	13	13	11
DCS visual memory (sum of trials 1–5)	31	31	—	34
Picture naming				
Boston naming test (raw score)	56	—	54	54
Visuoconstruction				
WAIS‐IV Block design (scaled score)	15	—	15	15
Depression Screening				
Beck Depression Inventory—II (sum score)	2	—	—	3

*Note*: In bold: below average (±1 SD) based on the norms given by the test handbook.

Abbreviations: DCS, Diagnosticum fuer Cerebralschaedigung; MMSE, Mini‐Mental State Examination; RWT, Regensburger Wortfluessigkeitstest (Regensburg word fluency test); VLMT, Verbal Learning and Memory Test (German version of the AVLT = Auditory Verbal Learning Test); WAIS‐IV, Wechsler Adult Intelligence Scale – Fourth Edition; WMS‐R, Wechsler Memory Scale‐Revised.
